# Effect of Lee Silverman Voice Treatment-BIG Intervention Versus Balance Training via Tele-rehabilitation on Balance and Functional Mobility Among Community-Dwelling Elderly: A Comparative Study

**DOI:** 10.7759/cureus.103965

**Published:** 2026-02-20

**Authors:** Meghana P Chavan, Priti N Agni

**Affiliations:** 1 Neurophysiotherapy, K J Somaiya College of Physiotherapy, Mumbai, IND

**Keywords:** age and ageing, balance training in geriatrics, community-dwelling elderly, functional mobility, lee silverman voice treatment big (lsvt big), tele-rehabilitation

## Abstract

Introduction: Elderly individuals often experience loss of confidence, gait abnormalities, and impaired balance, which can lead to fear of falling and limitations in functional mobility. Lee Silverman Voice Treatment-BIG (LSVT-BIG) addresses balance and activities of daily living (ADLs) in individuals with Parkinson’s disease by training them to increase the amplitude of movements from head to toe. It targets functional mobility through “functional component movements,” in which a specific task is selected and the individual is trained to perform it with large-amplitude movements and then apply it to real-life activities. LSVT-BIG has been shown to improve balance and mobility in Parkinson’s disease, which are also features of the "ageing syndrome." Hence, this study aims to explore its role in the geriatric population, compared with traditional balance training methods, through tele-rehabilitation for balance and functional mobility.

Materials and methods: This comparative study was conducted on 34 community-dwelling elderly participants in Mumbai, Maharashtra, India. Participants were screened based on the inclusion criteria and allocated into two equal groups. Group A (n = 17) received the LSVT-BIG protocol, while Group B (n = 17) received conventional balance training. Both interventions were delivered via tele-rehabilitation for four consecutive days per week over a period of four weeks. Pre- and post-intervention assessments were performed using the Mini-Balance Evaluation Systems Test (Mini-BESTest) to assess balance and the Timed Up and Go (TUG) test to assess functional mobility.

Results: Intra-group analysis showed a statistically significant difference between pre- and post-intervention values for both the Mini-BESTest and TUG in both groups (p < 0.0001). There was no statistically significant difference between Groups A and B in Mini-BESTest scores (p = 0.2622). However, a statistically significant difference was observed between the TUG scores of Groups A and B (p = 0.0066).

Conclusion: Both LSVT-BIG and conventional balance training delivered through tele-rehabilitation significantly improved balance and functional mobility in the elderly population. Compared to conventional balance training, LSVT-BIG demonstrated superior improvement in functional mobility.

## Introduction

In India, there is a rapid increase in the elderly population, which is expected to reach approximately 326 million by 2050. This trend raises increasing concern regarding various socioeconomic and health-related issues [1]. With advancing age, individuals face challenges in maintaining independence and self-confidence while performing activities of daily living (ADLs), despite higher rates of functional decline and disability observed among community-dwelling elderly individuals [2]. ADLs require multiple components of balance, and impairment in these components progressively compromises functional mobility and quality of life (QOL). During normal ageing, physiological changes in the multisensory system affect balance and lead to more fearful movement patterns, thereby influencing the efficiency of functional task performance. Age-related changes in bone density, cartilage integrity, joint flexibility, and proprioception contribute to increased musculoskeletal stiffness and weakness, adversely affecting balance [3]. Additionally, increased displacement and velocity of the center of pressure (CoP) are observed due to poor postural control during tasks requiring both static and dynamic balance. With advancing age, disruption of the sensorimotor control system further affects postural control and balance [4]. This results in impaired mobility and induces a psychological fear of falling, which in turn leads to reduced participation in ADLs among older adults [5]. Therefore, balance training and conventional exercise programs play an important role in improving postural control by enhancing lower-limb strength and joint proprioception, thereby reducing the risk of falls.

Lee Silverman Voice Treatment-BIG (LSVT-BIG) is an intervention originally developed for individuals with Parkinson’s disease (PD) and is derived from the speech treatment LSVT-LOUD, which is used to manage speech deficits such as hypophonia [6]. LSVT-BIG addresses balance and ADLs in PD by training individuals to increase the amplitude of movements from head to toe. It also targets functional mobility through “functional component movements,” wherein a specific task is selected and the individual is trained to perform it using large-amplitude movements, followed by retraining in real-life activities [6]. LSVT-BIG incorporates key principles of motor learning, including high intensity, salience, multiple repetitions, and progressive complexity. These principles facilitate activity-dependent motor learning and neuroplasticity, thereby enhancing movement generalization and automaticity. LSVT-BIG has shown benefits in stroke rehabilitation, and researchers are exploring its application in other neurological conditions [7]. While several interventions such as Tai Chi and various forms of yoga have been explored to improve balance and functional mobility in the elderly, LSVT-BIG may also be effective in addressing balance and mobility impairments seen in Parkinson’s disease, which share similarities with manifestations of the ageing syndrome [7,8]. Hence, this study aims to examine the role of LSVT-BIG in geriatrics and its effects on balance and functional mobility, which are common features of ageing.

Tele-rehabilitation has emerged as a valuable approach in the field of rehabilitation, offering time- and cost-effective healthcare services, particularly for vulnerable populations such as the elderly and individuals with disabilities [9]. It has been utilized in the rehabilitation of various conditions, including stroke, multiple sclerosis, cardiorespiratory disorders, and psychological conditions, and has demonstrated effectiveness in improving quality of life, functional abilities, and reducing long-term symptoms [10]. However, there is limited literature on the effectiveness of tele-rehabilitation in improving balance and functional mobility among community-dwelling elderly individuals.

Thus, this study contributes to the existing literature by examining the role of balance training delivered through tele-rehabilitation in the geriatric population, while also exploring LSVT-BIG as a potential intervention to improve balance and functional mobility in the elderly. The study holds social relevance by promoting healthy ageing and enhancing community participation among older adults.

## Materials and methods

This was a comparative study conducted over a period of 18 months among community-dwelling, ambulatory adults aged 65-80 years in a tertiary care physiotherapy outpatient department and community setting. The study was conducted in accordance with the ethical principles outlined in the Declaration of Helsinki. Institutional ethical approval was obtained prior to study commencement, and written informed consent was obtained from all participants. The study was reported in accordance with the Strengthening the Reporting of Observational Studies in Epidemiology (STROBE) guidelines.

Participants were screened based on predefined inclusion and exclusion criteria. Inclusion criteria comprised community-dwelling elderly individuals who were not engaged in structured exercise programs, were accessible for tele-rehabilitation, and had caregiver support during intervention sessions. Exclusion criteria included individuals with medical, neurological, or sensory conditions limiting exercise participation; those with high or minimal fall risk (Mini -Balance Evaluation Systems Test (Mini-BESTest) score <19.5 or ≥28; Timed Up and Go (TUG) test >13.5 seconds); and individuals unwilling to participate.

Sample size was calculated using the OpenEpi website (Dean AG, Sullivan KM, Soe MM. OpenEpi: Open Source Epidemiologic Statistics for Public Health, Version. www.OpenEpi.com, updated 2013/04/06), with a 95% CI and 80% power, accounting for a 10% attrition rate. The required sample size was determined to be 17 participants per group (50%), resulting in a total sample size of 34 participants (100%). Participants were equally allocated to two intervention groups with comparable baseline characteristics. 

**Figure 1:**
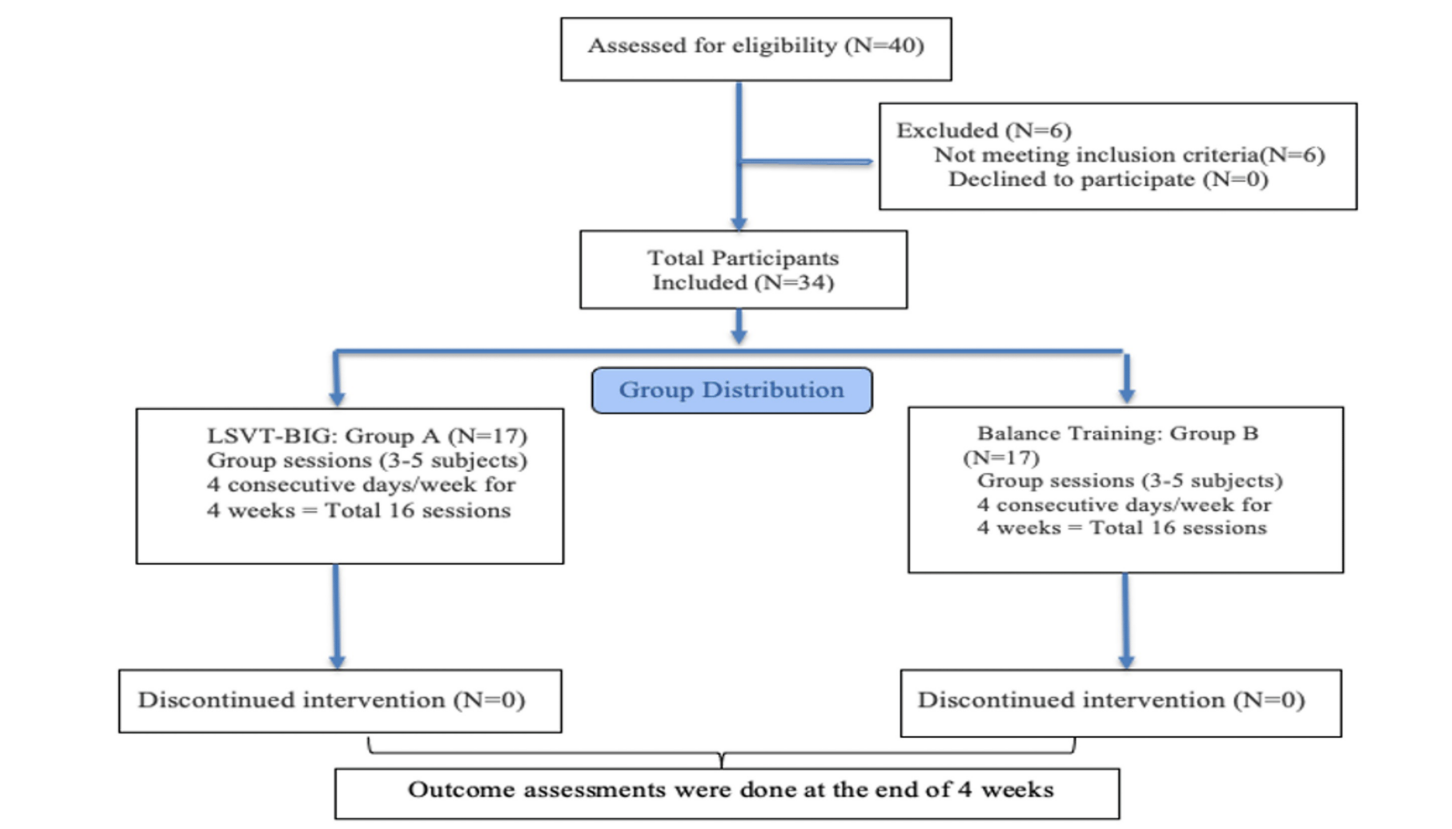
Participant flow diagram

**Figure 2:**
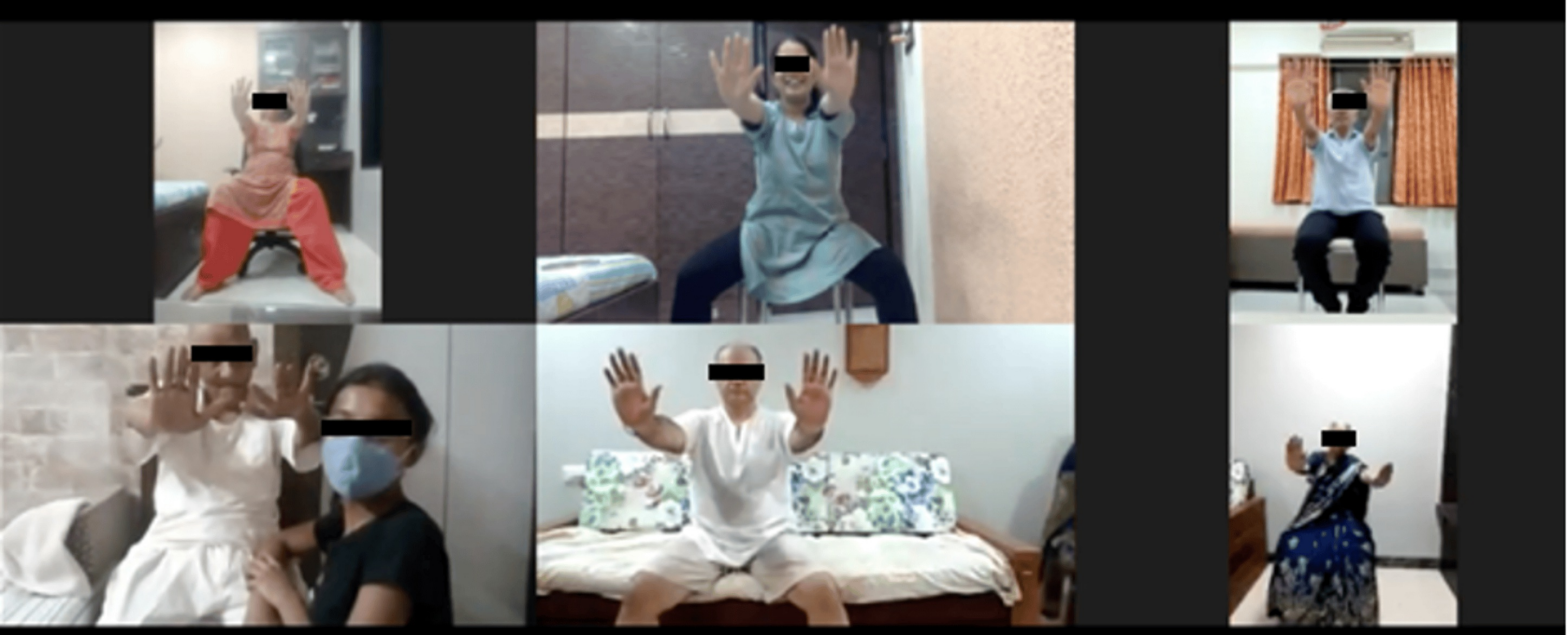
Therapist’s screen while conducting the session through tele-rehabilitation

Figure 1 depicts the study design and participant flow throughout the research process. The study received approval from the Institutional Ethics Committee (IEC), and participants were recruited using convenience sampling from the community. The study procedure was explained in the participants’ preferred language, and informed consent was obtained. Eligible participants were assigned to either Group A (Tables 1, 2) or Group B (Tables 3, 4), with 17 participants in each group. Both interventions were delivered via tele-rehabilitation in small groups. Outcome assessments were conducted offline by the same assessor before and after the intervention to ensure consistency. Balance was assessed using the Mini-BESTest, and functional mobility was assessed using the TUG test. The therapist administering the interventions was certified in the LSVT-BIG method and provided the respective interventions accordingly. During tele-rehabilitation sessions (Figure 2), participants were instructed to exercise in a safe home environment near fixed support surfaces to minimize the risk of falls and were accompanied by a family member for safety. Family members were briefed on the exercise protocol, and instructional videos were provided prior to the sessions. Attendance was monitored throughout the intervention period, and reminders were sent to promote adherence. Post-intervention assessments were conducted by the same assessor.

**Table 1:** Intervention dosage for group A

Group A	Intervention
Intervention	Lee Silverman Voice Treatment-BIG (LSVT-BIG)
Dosage	Four consecutive days per week for four weeks (16 sessions in one month)
Session duration	Sixty minutes per session
Repetitions	Minimum of 8-12 repetitions per task
Effort level	Maximum patient-perceived effort daily (8-9 on a 1-10 scale)

**Table 2:** Exercise protocol for group A

Session phase	Task	Exercise	Repetitions/duration
First half (45 min)	Task 1: maximum sustained movements (seated)	BIG stretch: floor to ceiling	8 reps, 10-sec hold
		BIG stretch: side to side	8 reps, 10-sec hold
	Task 2: repetitive/directional movements (standing)	Forward BIG step	16 reps (8 each leg)
		Sideways BIG step	16 reps (8 each leg)
		Backward BIG step	16 reps (8 each leg)
		Forward BIG rock and reach	20 reps (10 each leg)
		Sideways BIG rock and reach	20 reps (10 each leg)
	Task 3: functional component movements	Functional activity components	5 reps per component
Second half of session (15 min)	Walking component	BIG walking included as part of hierarchy tasks	

**Table 3:** Intervention dosage for Group B

Group B	Intervention
Intervention	Conventional balance training
Dosage	Four consecutive days per week for four weeks (16 sessions in one month)
Session duration	Sixty minutes per session
Repetitions	Minimum of 8-12 repetitions per task
Effort level	Maximum patient-perceived effort daily (8-9 on a 1-10 scale)

**Table 4:** Intervention for Group B: conventional balance training protocol

Session component	Duration	Activities/description
Warm-up	Five minutes	Stepping exercises: forward and lateral walking at a safe moderate speed. Raising both arms overhead and lowering. Neck exercises: trunk rotations
Training (conventional balance exercises)	Fifty minutes (3-4 min/activity)	Marching in place; standing with feet close together; walking forward 10 steps and pivoting 180°; alternate single-leg standing; sit-to-stand from chair with arms crossed; heel raises holding chair; tandem standing; back and side leg raises holding chair; walking forward, sideways, and backward; marching with high leg lift; tandem walking along a line; stepping in different directions; mini wall squats; obstacle walking
Cool-down	Five minutes	Forward walking at moderate speed. Joint mobility exercises: raising arms overhead and lowering
Exercise progression	Throughout training	Reducing base of support; closing eyes; increasing movement speed; using unstable surfaces (e.g., soft pillow)

## Results

Data from 34 participants were collected, recorded in a Microsoft Excel spreadsheet (Microsoft Corp., Redmond, WA, USA), and analyzed using GraphPad Prism software version 9.4.0 (453) (Dotmatics, Boston, MA, USA). Qualitative variables were expressed as absolute numbers and percentages, while quantitative variables were expressed as mean and standard deviation. The Shapiro-Wilk test was used to assess the normality of data distribution (Tables 5, 6). For intra-group analysis, a paired t-test was used to compare pre- and post-intervention Mini-BESTest scores in Group A and Group B, and TUG scores in Group B. As the TUG scores in Group A did not follow a normal distribution, the Wilcoxon signed-rank test was used to compare pre- and post-intervention values in this group (Tables 7, 8). For inter-group analysis, an unpaired t-test was used to compare Mini-BESTest scores between Group A and Group B, while the Mann-Whitney U test was used to compare TUG scores between the two groups (Tables 9, 10). A p-value < 0.05 was considered statistically significant.

**Table 5:** Descriptive statistics for age distribution

Age (years)	Group A	Group B
Median	71	68
Mean	71.64	69.76
Standard deviation	5.43	5.48

**Table 6:** Descriptive statistics for gender distribution

Gender	Number of subjects		Percentage
	Group A	Group B	
Females	8	8	47
Males	9	9	53
Total	17	17	100

**Table 7:** Comparison of Mini-BESTest scores between the groups

Mini-BESTest scores		Mean	Std. deviation	N	p-value	Significance
Group A (LSVT-BIG)	Pre	23.17	1.74	17	<0.0001	Significant
	Post	25.05	1.56	17		
Group B (CBT)	Pre	22.82	1.74	17	<0.0001	Significant
	Post	24.35	1.76	17		

**Table 8:** Comparison of the Timed Up and Go scores between the groups

TUG scores		Mean	Std. deviation	N	p-value	Significance
Group A (LSVT-BIG)	Pre	11.52	1.06	17	<0.0001	Significant
	Post	9.88	0.92	17		
Group B (CBT)	Pre	10.76	1.30	17	0.0003	Significant
	Post	9.94	1.29	17		

**Table 9:** Between-group comparison of the Mini-BESTest scores

Mini-BESTest score	Mean	Std. deviation	p-value	Significance
Group A (LSVT-BIG)	1.88	0.85	0.2622	Not significant
Group B (CBT)	1.52	0.94		

**Table 10:** Between-group comparison of the TUG scores

TUG score	Mean	Std. deviation	p-value	Significance
Group A (LSVT-BIG)	-1.64	0.86	0.0066	Significant
Group B (CBT)	-0.82	0.72		

## Discussion

This is one of the pioneer studies to document and explore the effect of LSVT-BIG on balance and functional mobility in healthy elderly individuals through tele-rehabilitation. The age and gender distribution of participants in both groups were comparable, as shown in Table 5 and Table 6, indicating baseline similarity between the groups. The study demonstrated statistically significant intra-group improvements (Tables 7, 8) in Mini-BESTest and Timed Up and Go (TUG) scores in both Groups A and B. However, no statistically significant inter-group difference was observed in Mini-BESTest scores, as both groups showed comparable improvement in balance (Tables 9, 10).

The modest improvement in balance observed following LSVT-BIG may be attributed to repetitive, high-amplitude daily exercises that emphasize “BIG” movements, static posturing, and enhanced joint mobility, thereby reducing tissue rigidity. The use of exaggerated movements with strong distal sensory input (such as foot stomping and hand slapping) may facilitate sensory recalibration and sustained muscle activation, contributing to improved balance control. Previous work has shown that LSVT-BIG improves proprioceptive performance by recalibrating proprioceptive processing in individuals with PD [11]. In the present study, multidirectional movements such as forward, sideways, and backward BIG stepping, as well as forward rock and reach, may have positively influenced balance, which is commonly affected in community-dwelling elderly individuals due to age-related physiological changes.

LSVT-BIG also demonstrated statistically significant improvements in TUG scores, indicating a positive effect on functional mobility among the elderly. While prior studies have reported improvements in functional mobility following LSVT-BIG in individuals with PD, its effects had not been previously explored in a healthy elderly population [12]. The intervention includes dynamic exercises such as rock and reach, sideways rock and reach, and BIG walking. These exercises resemble normal gait patterns and require coordination between the upper and lower extremities, along with reciprocal ankle dorsiflexion and plantarflexion to facilitate a heel-to-toe walking pattern. The sideways rock and reach exercise is performed bilaterally and may contribute to improved truncal rotation. BIG walking emphasizes high-amplitude walking with increased arm swing and longer step lengths, aiming to override slow and reduced movement patterns [13,14]. BIG sit-to-stand was included as one of the functional component exercises relevant to activities of daily living. Both groups demonstrated statistically significant pre- to post-intervention improvements in balance and functional mobility.

The findings of this study are consistent with previous research examining the effects of functional balance training in the ageing population [3,4]. Studies have shown that functional balance training plays an important role in maintaining and improving functional abilities in older adults [8]. The improvements observed in the present study may be attributed to the multisensory approach adopted, incorporating visual, vestibular, and somatosensory inputs encountered during daily activities, thereby enhancing balance and functional mobility. Lower limb strengthening exercises such as sit-to-stand, wall squats, and heel raises likely contributed to improved functional mobility by enhancing lower limb muscle strength.

The results are also consistent with studies that implemented structured balance programs ranging from 4 to 25 weeks, involving positional variations, directional walking, and sensory challenges, which led to improvements in static and dynamic balance, functional mobility, and a reduction in fear of falling [3,4,8]. The present study, which combined offline assessments with online interventions, demonstrated statistically significant intra-group improvements, suggesting that both LSVT-BIG and conventional balance training delivered through tele-rehabilitation were effective in improving balance and functional mobility in community-dwelling elderly individuals.

A systematic review by Velayati et al. [9] examined the effectiveness of tele-rehabilitation in elderly individuals with chronic health conditions and highlighted the need for further research, as several studies reported outcomes comparable to conventional rehabilitation. This suggests that tele-rehabilitation may serve as an alternative or adjunct to traditional outpatient rehabilitation, reducing resource burden. Similarly, Tyagi et al. [15] concluded that tele-rehabilitation can be utilized for health promotion strategies in the elderly and as a complementary service delivery model. Thus, the present study adds to the growing body of literature supporting the effectiveness of LSVT-BIG delivered through tele-rehabilitation in improving balance and functional mobility in the elderly population.

Limitations

The study was limited by its restricted geographical scope, which may affect the generalizability of the findings. Additionally, age stratification was not performed due to unequal distribution across age groups. Outcome assessments were conducted only at pre- and post-intervention time points, with no follow-up to evaluate long-term or carry-over effects. Furthermore, subjective measures of quality of life were not included, despite the potential psychological benefits of the intervention. These aspects may be considered as future directions for research. 

## Conclusions

This study concludes that both LSVT-BIG and balance training delivered through tele-rehabilitation significantly improved balance and functional mobility in the elderly population. However, when compared to balance training alone, the LSVT-BIG program demonstrated superior and statistically significant improvements in functional mobility.
